# Protective Effects of Donkey Milk‐Derived Exosomes Against UVB‐Induced Skin Photoaging via Regulation of DNA Damage and Aging‐Related Pathways

**DOI:** 10.1155/bmri/4214259

**Published:** 2026-08-05

**Authors:** Jie Yu, Jinni Sun, Jie Cheng, Guangyuan Liu, Pengxiang Niu, Qian Liu, Zhaoting Wang, Ying Huo, Hang Tie, Cong Wang

**Affiliations:** ^1^ Dong-E E-Jiao Co. Ltd, Liaocheng, Shandong, China; ^2^ Tianjin Key Laboratory of Animal and Plant Resistance, College of Life Sciences, Tianjin Normal University, Tianjin, China, tjnu.edu.cn; ^3^ Chinese Academy of Inspection and Quarantine Greater Bay Area, Zhongshan, Guangdong, China

**Keywords:** DNA damage, donkey milk-derived exosomes, photoaging, senescence, UVB

## Abstract

Prolonged exposure to UVB radiation accelerates skin photoaging by inducing DNA damage, promoting collagen degradation, and triggering cellular senescence. There is a growing interest in identifying effective natural interventions to prevent or mitigate these adverse effects. This study investigated the protective role and underlying mechanisms of donkey milk‐derived exosomes (DM‐Exos) against UVB‐induced skin aging using both in vitro skin cell models and in vivo mouse models. The results demonstrated that DM‐Exos significantly reduced UVB‐induced cellular senescence, as evidenced by decreased *β*‐galactosidase activity and downregulation of senescence‐associated genes. DM‐Exos also attenuated intracellular ROS production and reduced DNA damage markers (*γ*‐H2AX and 8‐OHdG) both in vitro and in vivo. Transcriptomic analysis further revealed that DM‐Exos mitigated UVB‐induced alterations in pathways related to cellular senescence, DNA damage, cell proliferation, and response to UV radiation, which were corroborated by qPCR validation of key aging‐related genes. These findings underscore the potential of DM‐Exos as a promising natural agent for protecting against UVB‐induced skin aging and maintaining overall skin health.

## 1. Introduction

Aging is a complex biological process characterized by the progressive decline of cellular and tissue functions, reduced regenerative capacity, and increased susceptibility to diseases. While aging affects all organ systems, skin aging is one of the most visible manifestations, resulting from both intrinsic (chronological) and extrinsic (environmental) factors [1, 2]. Intrinsically, skin aging is driven by genetic and metabolic changes, whereas extrinsic factors, particularly ultraviolet (UV) radiation. UV radiation accelerates the aging not only by reactive oxygen species (ROS) accumulation in the skin but also by directly causing DNA damage, characteristically introducing bulky adducts such as cyclobutane pyrimidine dimers (CPDs) and double‐stranded breaks (DSBs) [3]. If DNA lesions remain unrepaired, cells undergo cell cycle arrest and senescence, a state characterized by the secretion of senescence‐associated secretory phenotype (SASP) factors, which further promote chronic inflammation and ECM degradation [4, 5, 6]. These processes contribute to key features of photoaging, including collagen degradation, loss of skin elasticity, increased roughness, and wrinkle formation [7, 8]. Given the rising concern over skin damage caused by UV exposure, identifying effective interventions to mitigate photoaging has become an important research focus.

Donkey milk, a traditional dairy product, has recently gained attention for its nutritional and therapeutic potential in combating oxidative stress‐related aging [9, 10, 11]. Notably, recent studies have identified extracellular exosomes (30–150 nm in size) in donkey milk, which are believed to contribute significantly to its antioxidant activity [12]. Proteomic and transcriptomic analyses using 4D label‐free quantitative proteomics and Illumina sequencing have revealed that DM‐Exos contain 2293 proteins and 212 miRNAs, respectively [12, 13]. Given their unique composition, DM‐Exos have attracted growing interest for their potential in mitigating UVB‐induced skin aging.

Our previous study demonstrated that DM‐Exos can protect against UVB irradiation‐induced ferroptosis in skin cells [14]. Building on this finding, the present study is aimed at further investigating the protective effects of DM‐Exos against UVB‐induced skin photoaging using both in vitro and in vivo models. At the cellular level, a senescence‐specific detection assay confirmed that UVB exposure induces skin cell senescence; however, DM‐Exos mitigated this phenomenon in a concentration‐dependent manner. Additionally, transcriptomic analysis identified key differentially expressed genes (DEGs) and enriched pathways associated with cellular senescence, DNA damage, cell growth, and response to UV, which were further validated by qPCR analysis of aging‐related genes across the control, UVB‐treated, and UVB + DM‐Exos groups. At the animal level, histological analysis of Collagens I and VII expression via hematoxylin and eosin (H&E) staining and immunohistochemistry demonstrated that DM‐Exos reduce collagen degradation, supporting their role in repairing UVB‐induced skin damage. These findings provide insight into DM‐Exos′ potential as a natural intervention for preventing UVB‐induced skin aging and highlight its broader implications for skin health and antiaging therapies.

## 2. Materials and Methods

### 2.1. Isolation and Characterization of Donkey Milk‐Derived Exosomes

Donkey mature milk (30 days postpartum) sample was first centrifuged at 2000 g for 30 min at 4°C, followed by further centrifugation of the supernatant at 10,000 g for 45 min at 4°C. The collected supernatant was filtered through a 0.45‐*μ*m membrane, and the resulting filtrate was ultracentrifuged at 100,000 × g for 70 min at 4°C. After discarding the supernatant, the pellet was resuspended in 1 mL of ice‐cold 1× PBS. This suspension was layered onto a 30% sucrose cushion (250 *μ*L) and ultracentrifuged again at 100,000 × g for 70 min. The interphase fraction (above the sucrose cushion) was collected and subjected to another ultracentrifugation at 100,000 × g for 70 min. Finally, the supernatant was discarded, and the exosome pellet was resuspended in 500 *μ*L PBS for storage at −80°C. Exosomal morphology was observed using TEM, particle size distribution was analyzed by nanoparticle tracking analysis (NTA), and exosome protein concentration was quantified using the BCA assay.

### 2.2. Cell Culture

The HaCaT human keratinocyte cell line, purchased from Tianjin Shewei Biotechnology Co. Ltd. (Tianjin, China), was cultured in MEM medium (Gibco, Carlsbad, California, United States) supplemented with 15% fetal bovine serum (FBS; ExCell, Suzhou, China) and 1% penicillin–streptomycin (Gibco). Cells were maintained at 37°C in a humidified atmosphere of 95% air and 5% CO_2_.

### 2.3. UVB‐Induced Skin Photoaging Model in Mice

Eight‐week‐old female Kunming mice (*Mus musculus*) were obtained from the Chengdu Dashuo Experimental Animal Company (Chengdu, China). The mice were housed under standard specific pathogen‐free (SPF) conditions with a 12‐h light/12‐h dark cycle, controlled temperature of 22°C ± 2°C, and relative humidity of 50% ± 10%. A UVB‐induced skin photoaging model was established as previously described [15, 16]. Briefly, mice were exposed to UVB radiation at escalating doses: 200 mJ/cm^2^ for Weeks 1–2, 400 mJ/cm^2^ for Weeks 3–4, and 600 mJ/cm^2^ for Weeks 5–6. Immediately following each UVB exposure, 20 *μ*g of DM‐Exos and MSC‐Exos were administered via subcutaneous injection into the dorsal skin of mice three times per week, representing a therapeutic (postexposure repair) regimen. Normal control mice received subcutaneous injections of the same volume of saline on the same schedule. Experimental mice were deeply anesthetized with tribromoethanol (250 mg/kg, i.p.) prior to euthanasia by sacrificing.

### 2.4. UVB‐Induced Cellular Senescence Model

HaCaT cells were divided into three groups: a control group, a UVB‐treated group, and a UVB + DM‐Exos repair group. After cells adhered to the culture plates overnight in the incubator, the culture medium was replaced with 1 mL of PBS to maintain the cells′ hydration. The dish covers were then removed to expose the cells to UVB irradiation. The irradiation dose was set at 20 mJ/cm^2^ per day and DM‐Exos for seven consecutive days, resulting in a total cumulative dose of 140 mJ/cm^2^. The irradiation time was adjusted based on the distance between the UVB lamp and the cells to ensure consistent exposure.

### 2.5. *β*‐Gal Staining

*β*‐gal staining was performed using the senescence‐associated *β*‐gal staining kit (Beyotime, Shanghai, China) according to the manufacturer′s instructions. Briefly, HaCaT cells were seeded into six‐well plates and cultured for 24 h. At 72 h after the final UVB irradiation, the culture medium was removed, and cells were gently washed once with PBS. Cells were then fixed with the fixation solution provided in the kit for 15 min at room temperature. Following fixation, cells were washed three times with PBS, 3 min each time. Excess PBS was removed, and 1 mL of freshly prepared *β*‐gal staining working solution from the kit was added to each well. Plates were incubated overnight at 37°C in a humidified chamber without CO_2_. After incubation, cells were examined under a light microscope, and representative images were captured for analysis.

### 2.6. Quantitative Real‐Time PCR

Total RNA was extracted from cells or tissues using TRIzol reagent (Beyotime, Shanghai, China), and concentrations were determined with a Nanodrop Spectrophotometer (Thermo Fisher Scientific, United States). cDNA synthesis was performed using the HiScript III RT SuperMix for qPCR (Vazyme, Nanjing, China), and gene expression was quantified using ChamQ Universal SYBR qPCR Master Mix (Vazyme, Nanjing, China). The thermal cycling conditions were as follows: initial denaturation: 95°C for 2 min; amplification: 40 cycles of 95°C for 10 s, and 60°C for 30 s; dissociation curve analysis: 95°C for 15 s, 60°C for 1 min, 95°C for 1 min, and 55°C for 15 s. All reactions were performed in triplicate, and relative gene expression was normalized to *β*‐actin using the 2^-*ΔΔ*Ct^ method. The primers for H2AC6 were purchased from Beyotime (QH25997S and QM69778S). Other primer sequences are provided in Table S1.

### 2.7. Immunohistochemical (IHC) Staining

Mouse skin tissues were harvested and fixed overnight at 4°C in 4% paraformaldehyde (PFA) prepared in 10‐mM PBS. After paraffin embedding, 5‐*μ*m–thick skin sections were cut using a paraffin microtome. The sections were then deparaffinized using routine procedures, followed by antigen retrieval using an alkaline retrieval solution in a pressure cooker for 2 min. To block endogenous peroxidase activity, tissue sections were washed with PBS for 5 min, then incubated with 0.3% H_2_O_2_ in PBS for 10 min at 25°C. After another 5‐min PBS wash, sections were incubated with 10% normal goat serum for 30 min at room temperature to block nonspecific binding. Sections were then incubated overnight at 4°C with primary antibodies against COL1A1 (1:2000, HuaAn Biotechnology Co. Ltd., Hangzhou, China) and COL7A1 (1:2000, Qinke Biological Research Center Co. Ltd., Jiangsu, China). The following day, sections were incubated with HRP‐labeled goat antirabbit IgG (1:500) for 2 h. After washing, DAB substrate was applied for 2 min at room temperature to visualize staining. Tissue sections were then counterstained with hematoxylin for 40 s and dehydrated through a graded alcohol series.

### 2.8. H&E Staining

Experimental mice were euthanized by decapitation, and the dorsal skin was carefully harvested and cut into appropriate sizes. Skin samples were fixed in 10% formalin for 48 h, followed by dehydration and paraffin embedding. Paraffin‐embedded samples were sectioned at 4 *μ*m, mounted on slides, and stained with H&E. Sections were deparaffinized in xylene after a 30 s wash with distilled water. Finally, slides were sealed with neutral resin, examined under a microscope, and photographed.

### 2.9. Measurement of Skin Hydration and Roughness

Skin samples measuring 6 mm in diameter were extracted from the dorsal back. Hydration and elasticity of the dorsal skin surface were measured with the Mericom Hydration Meter model 1504.

### 2.10. Sample Quality Control, Library Preparation, and Sequencing

Total RNA integrity was assessed using the RNA Nano 6000 Assay Kit on the Agilent Bioanalyzer 2100 system. Polyadenylated mRNA was purified using poly‐T oligo‐attached magnetic beads, fragmented using divalent cations in First Strand Synthesis Reaction Buffer (5X), and reverse transcribed into first strand cDNA using random hexamer primers and M‐MuLV Reverse Transcriptase. Second strand cDNA was synthesized using DNA polymerase I and RNase H. After end repair, A‐tailing, and adaptor ligation, 370–420 bp cDNA fragments were selected using the AMPure XP system, followed by PCR amplification with Phusion High‐Fidelity DNA polymerase, Universal PCR primers, and Index primers. Library quality was assessed using the Agilent Bioanalyzer 2100. Clustering was performed on the cBot system using the TruSeq PE Cluster Kit v3‐cBot‐HS, and libraries were sequenced on the Illumina NovaSeq platform, generating 150 bp paired‐end reads.

### 2.11. Read QC, Alignment, Mapping, Gene Quantification, Normalization

Raw sequencing reads were quality‐filtered using FastQC and trimmed using Trimmomatic (or another trimming tool) to remove adapter sequences and low‐quality bases. Clean reads were aligned to the human genome (GRCh38/hg38) using HISAT2 software. Aligned reads were quantified using featureCounts, producing a gene‐level count matrix used for subsequent differential expression analysis.

### 2.12. Differential Expression Analysis

To identify differences between the UVB‐irradiated group and the control group, as well as between the UVB‐irradiated group and the DM‐EXOS repair group. Utilizing the DESeq2 R package (1.46.0). DESeq2 provides statistical routines for determining differential expression in digital gene expression data using a model based on the negative binomial distribution. The resulting *p* values were adjusted using the Benjamini and Hochberg′s approach for controlling the false discovery rate. Genes with an adjusted *p* value ≤ 0.05 and an absolute fold change (|*f* *o* *l* *d* *c* *h* *a* *n* *g* *e*|) exceeding 0.263 found by DESeq2 were assigned as differentially expressed.

Differential expression analysis between the UVB‐irradiated and control groups, as well as between the UVB‐irradiated and UVB + DM‐EXOS groups, was performed using the DESeq2 package in R. Gene expression levels were normalized using DESeq2′s default method (median‐of‐ratios method) prior to differential expression analysis. Genes with an adjusted *p* value < 0.05 (adjusted using Benjamini–Hochberg correction to control false discovery rate) and an absolute log2 fold‐change (|*f* *o* *l* *d* *c* *h* *a* *n* *g* *e*|) > 0.263 were identified as differentially expressed.

### 2.13. Gene Ontology (GO) and KEGG Enrichment Analysis of DEGs

GO and KEGG enrichment analyses of DEGs were performed using the clusterProfiler R package (v4.12.6). Human gene annotations were obtained from the org.Hs.eg.db database, and gene length bias was corrected. GO terms and KEGG pathways with a corrected *p* value < 0.05 were considered significantly enriched.

### 2.14. Statistical Analysis

Statistical analyses were performed using GraphPad Prism 8.0. For comparisons among three or more groups, one‐way ANOVA was performed followed by Tukey′s honest significant difference (HSD) post hoc test for multiple comparisons. For comparisons between two groups only, an unpaired *t*‐test was conducted. All data are presented as mean ± SEM from at least three independent experiments. Statistical significance thresholds were established in the figures by the bars and asterisks: ∗*p* < 0.05, ∗∗*p* < 0.01, ∗∗∗*p* < 0.001, and ∗∗∗∗*p* < 0.0001, with “ns” indicating nonsignificant results.

## 3. Results

### 3.1. Donkey Milk‐Derived Exosomes Ameliorate UVB‐Induced Damage in Skin Cells

**Figure 1:**
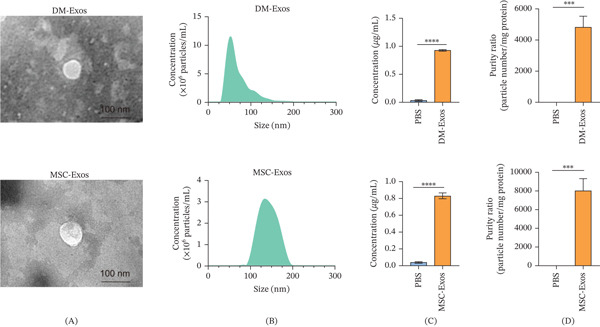
Extraction and characterization of exosomes from donkey milk. (A) Representative TEM images of DM‐Exos and MSC‐Exos. (B) Particle size distribution of exosomes as determined by NanoFCM. (C) Box plots of protein concentration in DM‐Exos and MSC‐Exos. (D) Purity assessment of Exos by particle‐to‐protein ratio.

Initial characterization confirmed the successful isolation of biologically active exosomes from donkey milk. Transmission electron microscopy revealed typical cup‐shaped vesicles (40–100 nm) with intact lipid bilayers, and the protein concentration was determined to be 1 *μ*g/*μ*L (Figure 1A,C). To further assess exosome purity, we calculated the particle‐to‐protein ratio as previously described [17]. As shown in Figure 1D, DM‐Exos exhibited a particle‐to‐protein ratio of approximately 5 × 10^7^ particles/*μ*g protein, comparable with that of MSC‐Exos, indicating high exosome purity.

**Figure 2:**
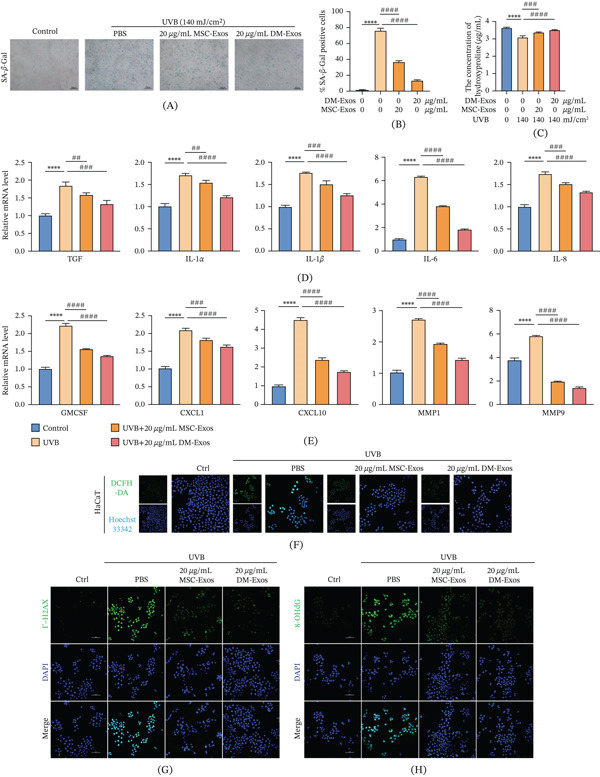
DM‐Exos alleviated UVB‐induced cellular senescence and suppressed SASP expression in HaCaT cells. (A–B) Representative SA‐*β*‐gal staining images of HaCaT cells in control, UVB‐treated, UVB+20 *μ*g/mL MSC‐Exos, and UVB+20 *μ*g/mL DM‐Exos groups. Scale bar = 50 *μ*m. (C) Hydroxyproline levels in HaCaT cells across the indicated experimental groups. (D–E) qPCR analysis of mRNA expression of SASP‐associated factors in HaCaT cells under each treatment condition. (F) Representative fluorescence images of DCFH‐DA staining showing intracellular ROS levels in HaCaT cells. (G–H) Immunofluorescence staining of *γ*‐H2AX and 8‐OHdG in HaCaT cells under control, UVB, UVB + MSC‐Exos, and UVB + DM‐Exos conditions.

To further elucidate the protective effects of DM‐EXOS on UVB‐induced skin senescence, we established an in vitro model of UVB‐induced damage using HaCaT cells irradiated with 140 mJ/cm^2^ UVB. Mesenchymal stem cell–derived exosomes (MSC‐Exos) were used as a positive control due to their well‐established radioprotective properties against UVB [18]. Subsequently, the UVB‐irradiated HaCaT cells were treated with DM‐Exos and MSC‐Exos at concentrations of 20 *μ*g/mL, followed by *β*‐galactosidase activity staining. As depicted in Figure 2A,B, the *β*‐galactosidase‐positive rate in UVB‐irradiated HaCaT cells was significantly elevated, nearly 80 times compared with the control group. Notably, DM‐Exos treatment resulted in a more pronounced reduction in the SA‐*β*‐gal–positive rate compared with MSC‐Exos treatment, suggesting that DM‐Exos are more effective in alleviating UVB‐induced cellular senescence.

Collagen synthesis is crucial for maintaining skin structure, elasticity, and firmness, and its impairment is a hallmark of skin aging [19]. To evaluate changes in collagen content, hydroxyproline—a major component of collagen—was measured in HaCaT cells. As illustrated in Figure 2C, UVB irradiation significantly reduced hydroxyproline levels about 20% compared with the control group, indicating impaired collagen synthesis. In contrast, DM‐Exos treatment led to a slight increase in hydroxyproline levels in UVB‐irradiated cells relative to MSC‐Exos treatment, further supporting its role in preserving collagen integrity.

Senescent cells secrete various cytokines, chemokines, growth factors, and matrix metalloproteinases (MMPs), collectively referred to as SASP [20]. To further investigate this effect, we assessed the mRNA expression levels of key SASP‐related factors, including TGF‐*β*, IL‐1*α*, IL‐1*β*, IL‐6, IL‐8, p21, GM‐CSF, CXCL1, CXCL10, MMP1, and MMP9. UVB irradiation significantly upregulated these markers, whereas MSC‐Exos and DM‐Exos treatment reversed these changes in a dose‐dependent manner, with DM‐Exos treatment exhibiting a more pronounced restorative effect (Figure 2D–E).

To directly assess whether DM‐Exos attenuate UVB‐induced oxidative stress, we performed DCFH‐DA staining to measure intracellular ROS levels in HaCaT cells. As shown in Figure 2F, UVB irradiation significantly increased ROS fluorescence intensity, which was markedly reduced by Exos treatment. We next examined DNA damage and oxidative DNA damage by immunofluorescence staining for *γ*‐H2AX and 8‐OHdG in HaCaT cells. As presented in Figure 2G–H, UVB exposure strongly increased the fluorescence intensity of both markers, whereas DM‐Exos treatment significantly attenuated these signals, with a greater effect than MSC‐Exos. Together, these results demonstrate that DM‐Exos effectively counteract UVB‐induced cellular senescence by reducing SA‐*β*‐gal activity, alleviating ROS production, and mitigating DNA damage (as indicated by *γ*‐H2AX and 8‐OHdG), while also more potently attenuating the proinflammatory compared with MSC‐Exos.

### 3.2. Transcriptomic Analysis Identifies Pathways and Genes Associated With DM‐Exos–Mediated Protection Against UVB‐Induced Skin Damage

**Figure 3:**
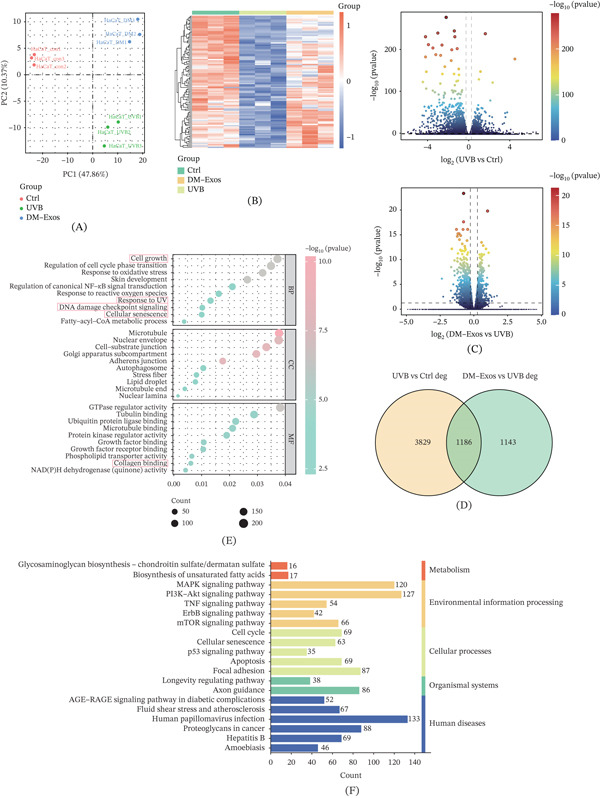
Transcriptomic analysis revealed that DM‐Exos protected against UVB‐induced skin cell damage by modulating DNA damage and ECM‐related pathways. (A) PCA plot illustrating distinct transcriptomic profiles of HaCaT cells among control, UVB‐treated, and UVB + DM‐Exos–treated groups. (B) Heatmap displaying DEGs among the three experimental groups. (C) Volcano plots of DEGs between UVB versus control (left panel) and UVB + DM‐Exos versus UVB (right panel). (D) Venn diagram showing overlapping DEGs between UVB versus control and UVB + DM‐Exos versus UVB comparisons. (E) GO enrichment analysis of DEGs across biological process, cellular component, and molecular function categories. (F) KEGG pathway enrichment analysis of DEGs. Dot size corresponds to the number of genes, and color represents the significance level.

To investigate the molecular mechanisms through which DM‐Exos protect against UVB‐induced skin damage, RNA sequencing (RNA‐seq) was performed on HaCaT cells divided into three groups: control, UVB‐treated, and UVB‐treated followed by DM‐Exos administration. Principal component analysis (PCA) revealed distinct separation among these groups, confirming the reproducibility of the experimental conditions and notable transcriptomic divergence due to UVB irradiation and DM‐Exos treatment (Figure 3A). DEGs were identified by comparing UVB‐treated versus control cells, as well as DM‐Exos–treated versus UVB‐treated cells. A heatmap illustrated expression patterns of the top identified DEGs, highlighting clear differences between the control and UVB groups, with DM‐Exos treatment partially reversing these transcriptomic alterations (Figure 3B). The volcano plots further visualized these gene expression changes, clearly displaying significant DEGs induced by UVB exposure and subsequently modulated by DM‐Exos treatment (Figure 3C). A Venn diagram analysis identified 1186 DEGs shared between UVB versus control and DM‐Exos + UVB versus UVB comparisons, suggesting these genes were specifically modulated by DM‐Exos intervention in response to UVB‐induced damage (Figure 3D). These 1186 overlapping genes were used for subsequent analyses. GO enrichment analysis showed that these DEGs were significantly involved in biological processes related to response to UVB radiation, DNA damage checkpoint signaling, cellular senescence, and collagen binding (Figure 3E). KEGG pathway enrichment further indicated significant involvement in critical signaling pathways, including p53 signaling, MAPK signaling pathway, PI3K‐Akt signaling pathway, and cellular senescence pathways (Figure 3F), highlighting the multidimensional protective role of DM‐Exos. Collectively, these transcriptomic data suggest that DM‐Exos may protect against UVB‐induced skin cell damage by modulating pathways associated with DNA damage, cellular senescence, and extracellular matrix (ECM) integrity.

### 3.3. Donkey Milk‐Derived Exosomes Alter the Expression of Aging‐Related Genes Induced by UVB Exposure

**Figure 4:**
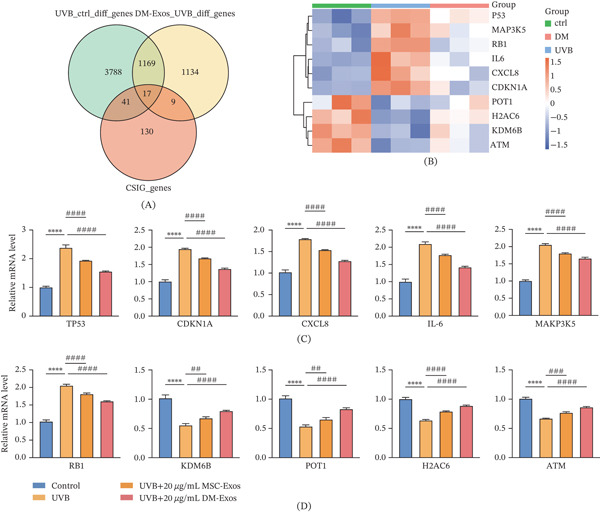
DM‐Exos reversed UVB‐induced changes in aging‐related gene expression. (A) Venn diagram showing the overlap among DEGs (UVB vs. control; UVB + DM‐Exos vs. UVB) and established aging‐related genes from the CSIG database. (B) Heatmap of expression levels for the top 10 overlapping aging‐related genes. (C–D) qPCR validation of selected aging‐related genes in HaCaT cells under different treatment conditions: control, UVB, UVB + 20 *μ*g/mL MSC‐Exos, and UVB + 20 *μ*g/mL DM‐Exos.

To determine whether DM‐Exos modulate aging‐related genes altered by UVB exposure, we intersected the RNA‐seq–derived DEGs with a curated set of aging‐associated genes obtained from the CSIG database (comprising 197 genes). This analysis identified 17 CSIG genes significantly modulated by both UVB exposure and subsequent DM‐Exos treatment (Figure 4A). Among these, 10 genes (p53, CDKN1A, CXCL8, IL6, MAP3K5, RB1, KDM6B, POT1, H2AC6, and ATM) were selected for further validation based on their biological relevance to cell cycle regulation, senescence, and ECM remodeling (Figure 4B). We then performed qPCR with HaCaT cells to independently confirm these findings (Figure 4C–D). Consistent with the transcriptomic data, UVB exposure markedly upregulated the expression of p53, CDKN1A (p21), CXCL8, IL6, MAP3K5, and RB1, whereas DM‐Exos treatment partially attenuated these increases. Conversely, UVB exposure downregulated the expression of KDM6B, POT1, H2AC6, and ATM, which were involved in telomere maintenance, histone modification, and DNA damage repair; DM‐Exos treatment partially restored their expression levels. These results indicate that DM‐Exos effectively regulates the expression of critical aging‐related genes dysregulated by UVB exposure, suggesting a protective role in reducing cellular senescence and associated molecular damage in skin cells.

### 3.4. Donkey Milk‐Derived Exosomes Ameliorate UVB‐Induced Skin Aging in Mice

**Figure 5:**
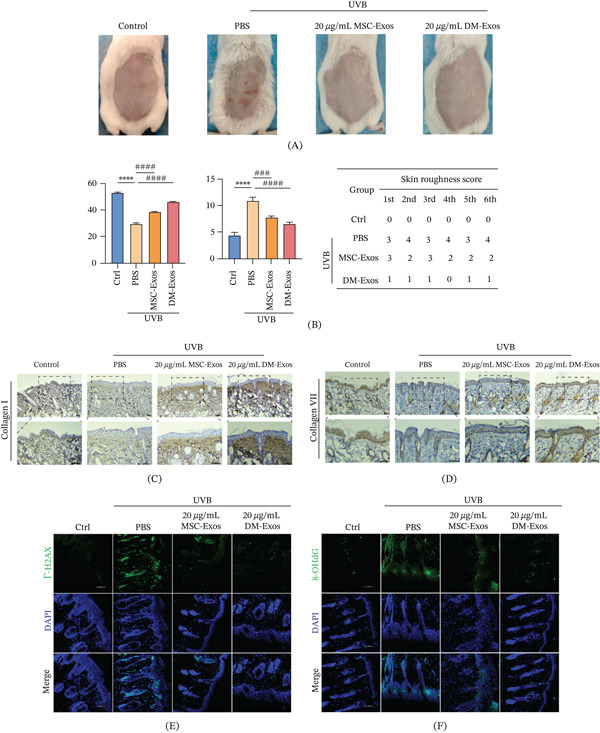
DM‐Exos ameliorated UVB‐induced skin photoaging in mice. (A) Representative images of dorsal skin in mice from the control, UVB, UVB + MSC‐Exos (100 *μ*g/kg), and UVB + DM‐Exos (100 *μ*g/kg) groups. (B) Quantitative measurements of skin moisture, elasticity, and roughness in mice across the indicated groups. (C, D) Immunohistochemical staining of (C) Type I collagen and (D) Type VII collagen in skin tissues from each experimental group. (E–F) Representative immunofluorescence staining images of *γ*‐H2AX and 8‐OHdG in mouse dorsal skin sections.

To assess the in vivo protective effects of DM‐Exos against UVB‐induced skin aging, we utilized a mouse model. Representative images of dorsal skin conditions (Figure 5A) revealed that UVB irradiation led to visible skin roughness, localized damage, and scabbing. In contrast, DM‐Exos treatment markedly improved the overall skin condition compared with the UVB‐exposed control. Key indicators of skin photoaging, including skin moisture content, elasticity, and roughness, were evaluated. Compared with the control group, UVB‐irradiated mice exhibited significantly reduced skin moisture content of about 45%, increased roughness, and diminished elasticity (Figure 5B). DM‐Exos treatment partially restored these parameters, highlighting its protective role against UVB‐induced skin photoaging.

Collagen is a key structural component of the skin, with Type I collagen providing strength and elasticity, whereas Type VII collagen supports epidermal–dermal adhesion and stability. To assess the impact of DM‐Exos treatment, we examined the expression levels of both collagen types using IHC staining. UVB exposure significantly reduced their expression compared with the control group (Figure 5C–D). However, DM‐Exos treatment restored collagen levels (Figure 5C–D). To further validate the protective effect of DM‐Exos against DNA damage in vivo, we performed immunofluorescence staining for *γ*‐H2AX and 8‐OHdG on mouse skin tissues. UVB irradiation induced strong positive staining for both markers, whereas DM‐Exos administration substantially reduced these signals (Figure 5E–F), indicating that DM‐Exos mitigates UVB‐induced DNA damage in vivo. These results demonstrate that DM‐Exos effectively protects against UVB‐induced skin aging in mice.

### 3.5. Donkey Milk‐Derived Exosomes Modulate UVB‐Induced Aging‐Related Gene Expression in Mouse Skin

**Figure 6:**
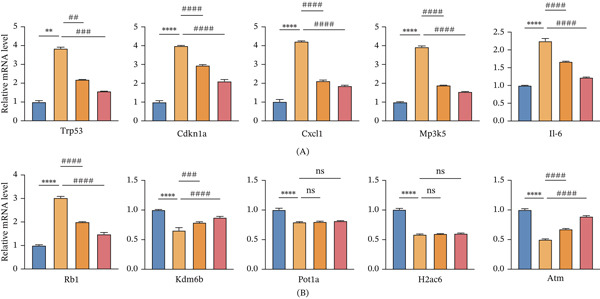
DM‐Exos ameliorated UVB‐induced alterations in aging‐related gene expression in mouse skin. (A–B) qPCR analysis of mRNA levels of selected aging‐related genes in mouse dorsal skin tissues from control, UVB‐treated, UVB + MSC‐Exos, and UVB + DM‐Exos groups.

**Figure 7:**
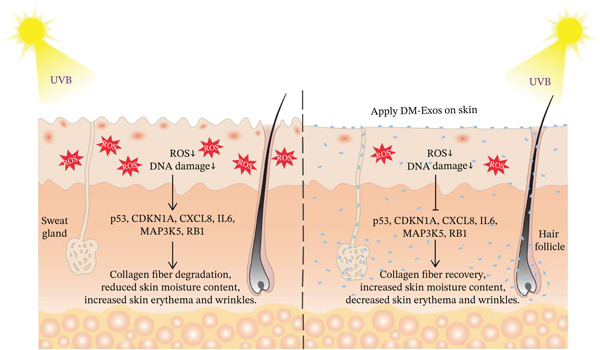
Proposed mechanism for the protective effect of DM‐Exos against UVB‐induced skin photoaging. UVB irradiation promoted ROS accumulation, leading to DNA damage and upregulation of aging‐related genes (e.g., p53, CDKN1A, CXCL8, IL6, MAP3K5, and RB1). These changes contributed to collagen degradation, decreased skin hydration, increased roughness, erythema, and wrinkle formation. DM‐Exos treatment attenuated UVB‐induced ROS generation and DNA damage, suppressed senescence‐associated gene expression, and helped maintain collagen structure. Collectively, these effects enhanced skin hydration and elasticity, while reducing erythema and wrinkle formation, highlighting the therapeutic potential of DM‐Exos in protecting and repairing UVB‐induced skin damage.

To further validate the protective role of DM‐Exos against UVB‐induced skin aging in vivo, we analyzed the expression of key aging‐related genes in mouse dorsal skin tissues. UVB irradiation significantly upregulated the expression of senescence‐ and inflammation‐associated genes, including p53, p21, CXCL8, IL6, and MAP3K5 (Figure 6A). DM‐Exos treatment partially reversed the UVB‐induced upregulation of these genes. Conversely, UVB exposure decreased the expression of genes related to epigenetic regulation, telomere maintenance, and DNA repair, including RB1, KDM6B, POT1, H2AC6, and ATM. DM‐Exos administration effectively restored their transcript levels toward baseline (Figure 6B). These in vivo findings corroborate the ability of DM‐Exos to counteract UVB‐driven molecular alterations linked to skin aging and underscore its promise as a natural therapeutic agent for mitigating photoaging (Figure 7).

## 4. Discussion

This study explored the protective effects of DM‐Exos against UVB‐induced skin aging using both in vitro and in vivo approaches. UVB exposure induced prominent cellular senescence, characterized by increased *β*‐galactosidase activity and elevated expression of SASP factors including IL6, CXCL8, MMP1, and MMP9. Treatment with DM‐Exos significantly attenuated these senescence markers, underscoring its potential to mitigate UVB‐induced cellular aging. Furthermore, RNA‐seq analysis revealed extensive gene expression alterations induced by UVB, predominantly involving pathways associated with DNA damage checkpoint signaling, cellular senescence, and collagen binding. Notably, several aging‐related genes, including p53, p21, IL6, MAP3K5, and RB1, exhibited significant expression changes following UVB exposure, indicative of a proaging molecular profile (Figures 2 and 4). Importantly, DM‐Exos intervention effectively reversed these UVB‐induced gene expression alterations, restoring their expression levels toward baseline and suggesting a protective mechanism involving the modulation of key cellular pathways implicated in skin aging.

Histologically, skin aging due to chronic UVB exposure is characterized by ECM degradation, particularly collagen loss, increased skin roughness, erythema, and wrinkle formation [15, 21]. Consistent with these observations, our in vivo experiments demonstrated significant degradation of collagen Types I and VII in the skin of UVB‐exposed mice, accompanied by reduced skin moisture content and diminished elasticity. Treatment with DM‐Exos markedly alleviated these histological and functional impairments, preserving collagen integrity and restoring key structural and physiological properties of the skin (Figure 5), thereby further supporting its therapeutic potential in combating photoaging.

Exosomes are nanoscale extracellular vesicles secreted by a wide variety of cell types, encapsulating diverse bioactive cargoes including proteins, lipids, and nucleic acids (such as mRNA, noncoding RNAs, and DNA). These vesicles are released by donor cells and can subsequently modulate the function of recipient cells [22, 23]. Exosomes are associated with aging‐related processes, including oxidative stress, inflammation, and cellular senescence [24, 25, 26, 27]. As reported, stem cell–derived exosomes can restore skin physiological function and regenerate or rejuvenate damaged skin tissue through various mechanisms such as decreased expression of MMP, increased collagen and elastin production, and modulation of intracellular signaling pathways as well as intercellular communication [28, 29].

We acknowledge a limitation of this study regarding exosome characterization. Due to the lack of donkey‐specific antibodies and limited sample availability, we were unable to perform Western blot or flow cytometry validation of canonical exosomal markers (e.g., CD9, CD63, and TSG101), as recommended by MISEV guidelines. However, our DM‐Exos preparations were characterized by TEM or particle‐to‐protein ratio and demonstrated consistent functional bioactivity across multiple assays. Future work with donkey‐specific reagents will be necessary to fully characterize the exosomal protein cargo.

In the present study, we extended these findings by exploring the protective role and underlying molecular mechanisms of DM‐Exos derived from donkey milk against UVB‐induced skin aging, utilizing both in vitro skin cell models and in vivo murine models. Transcriptomic profiling identified a panel of DEGs and enriched signaling pathways associated with cellular senescence, DNA damage response, cell proliferation, and UV response, further substantiating the antiaging properties of DM‐Exos.

We propose a working mechanistic model (Figure 7) illustrating how DM‐Exos confer protection against UVB‐induced skin aging. Chronic UVB exposure leads to excessive ROS accumulation, triggering DNA damage and the activation of aging‐associated signaling pathways—including upregulated expression of p53, p21, IL‐6, and MAP3K5—which collectively contribute to collagen degradation and impaired skin functionality. Topical application of DM‐Exos effectively attenuated ROS generation, reduced DNA damage, and restored the balanced expression of these critical aging‐related genes. Consequently, DM‐Exos treatment preserved collagen architecture, maintained skin hydration and elasticity, and alleviated the visible signs of cutaneous aging.

## 5. Conclusion

Our findings demonstrate that DM‐Exos exerts protective effects against UVB‐induced skin aging by reducing oxidative stress, attenuating cellular senescence, and preserving collagen synthesis and ECM integrity. Both transcriptomic and qPCR analyses identified specific molecular pathways—particularly those related to inflammation, ECM remodeling, and DNA repair—that are modulated by DM‐Exos treatment. Future investigations aimed at identifying the precise bioactive components within DM‐Exos that mediate these protective effects may facilitate the development of targeted therapeutic strategies for the prevention and treatment of photoaging and associated skin pathologies.

## Author Contributions

Jie Yu: investigation, methodology, writing—original draft. Jinni Sun: investigation, data curation. Jie Cheng: methodology, validation. Guangyuan Liu: formal analysis, validation. Pengxiang Niu: formal analysis, validation. Qian Liu: validation. Zhaoting Wang: software. Ying Huo: software. Hang Tie: conceptualization, supervision. Cong Wang: conceptualization, methodology, project administration, supervision, writing—review and editing.

## Funding

No funding was received for this manuscript.

## Ethics Statement

Animal studies were approved by the Institute Research Ethics Committee (No. 2023031303).

## Conflicts of Interest

Jie Yu, Jinni Sun, Jie Cheng, and Guangyuan Liu were employed by the Dong‐E E‐Jiao Co. Ltd. The other authors declare no conflicts of interest.

## Supporting information


**Supporting Information** Additional supporting information can be found online in the Supporting Information section. Table S1: Primer sequences used for quantitative real‐time PCR (qPCR) in this study. The table lists the gene names forward and reverse primer sequences.

## Data Availability

The data that support the findings of this study are available from the corresponding authors upon reasonable request.
