# Supplementary material for: Protective Effects of Donkey Milk‐Derived Exosomes Against UVB‐Induced Skin Photoaging via Regulation of DNA Damage and Aging‐Related Pathways
Source: Biomed Res Int. 2026 Aug 5;2026:4214259. doi: 10.1155/bmri/4214259 (PMC13443119; doi:10.1155/bmri/4214259)
Supplement: Supplementary file 1 [file BMRI-2026-4214259-s001.pdf]

Supporting Information

Table S1

Gene name		Primer Sequences
β-actin	Forward primer:	5’-AGCCTTCCTTCCTGGGCAT-3’
	Reverse primer:	5’-TGATCTTCATTGTGCTGGGTG-3’
TGF-β	Forward primer:	5’-CAAGTGGACATCAACGGGTTC-3’
	Reverse primer:	5’-GCAGCAGTTCTTCTCCGTGG-3’
IL-1α	Forward primer:	5’-AGTGCTGCTGAAGGAGATGC-3’
	Reverse primer:	5’-AGCACACCCAGTAGTCTTGC-3’
IL-1β	Forward primer:	5’-GCCCTAAACAGATGAAGTGCTC-3’
	Reverse primer:	5’-GAAGCCCTTGCTGTAGTGGT-3’
IL6	Forward primer:	5’-CCTTCTCCACAAGCGCCTTC-3’
	Reverse primer:	5’-TGGAATCTTCTCCTGGGGGT-3’
IL8	Forward primer:	5’-ACTCCAAACCTTTCCACCCC-3’
	Reverse primer:	5’-ATGAATTCTCAGCCCTCTTCAA-3’
p21	Forward primer:	5’-AGGGGACAGCAGAGGAAGAC-3’
	Reverse primer:	5’-CGTTTGGAGTGGTAGAAATCTGTC-3’
GMCSF	Forward primer:	5’-TTTGACCTCCAGGAGCCGAC-3’
	Reverse primer:	5’-ATCTGGGTTGCACAGGAAGT-3’
CXCL1	Forward primer:	5’-CCCAAACCGAAGTCATAGCCA-3’
	Reverse primer:	5’-TTGGATTTGTCACTGTTCAGCATC-3’
CXCL10	Forward primer:	5’-AGTGGCATTCAAGGAGTACCT-3’
	Reverse primer:	5‘-CGTGGACAAAATTGGCTTGC-3’
MMP1	Forward primer:	5’-CACGCCAGATTTGCCAAGAG-3’
	Reverse primer:	5’-GTTGTCCCGATGATCTCCCC-3’
MMP9	Forward primer:	5’-CTTTGAGTCCGGTGGACGAT-3’
	Reverse primer:	5’-TCGCCAGTACTTCCCATCCT-3’
TP53	Forward primer:	5’-AACAACACCAGCTCCTCTCC-3’
	Reverse primer:	5’-TTTGGACTTCAGGTGGCTGG-3’
MAP3K5	Forward primer:	5’-TACCGACTGGCTGAGAGTGA-3’
	Reverse primer:	5’-ACAGCATCCCTCCCCTTAGT--3’
RB1	Forward primer:	5’-CAGATGAAGCAGATGGAAGTAAACA-3’
	Reverse primer:	5’-TTCTGCTTTTGCATTCGTGTTC-3’
KDM6B	Forward primer:	5’-GCCTCTTCTCCACCAAGACC-3’
	Reverse primer:	5’-GCCTGGTACTGTGCGTACTT-3’
POT1	Forward primer:	5’-TCTAAGCAAAGGAACTGATTATTGC-3’
	Reverse primer:	5’-TCTTCAGCCTGTGAAAGCGAA-3’
ATM	Forward primer:	5’-TGCACTTCCGTCAGCAAAGA-3’
	Reverse primer:	5’-AAGGCTGCGCTTACACATCT-3’
mouse-actin	Forward primer:	5’-GTGACGTTGACATCCGTAAAGA-3’
	Reverse primer:	5’-GCCGGACTCATCGTACTCC-3’
mouse-p53	Forward primer:	5’-CCCCTGTCATCTTTTGTCCCT-3’
	Reverse primer:	5’-AGCTGGCAGAATAGCTTATTGAG-3’
mouse-p21	Forward primer:	5’-CCTGGTGATGTCCGACCTG-3’
	Reverse primer:	5’-CCATGAGCGCATCGCAATC-3’
mouse-Cxcl1	Forward primer:	5’-CTGGGATTCACCTCAAGAACATC-3’

	Reverse primer:	5'-CAGGGTCAAGGCAAGCCTC-3'
mouse-II6	Forward primer:	5'-CTGCAAGAGACTTCCATCCAG-3'
	Reverse primer:	5'-AGTGGTATAGACAGGTCTGTTGG-3'
mouse-Map3k5	Forward primer:	5'-GTTTCTGGAACGTGGAGAGC-3'
	Reverse primer:	5'-CTTCCCGAAATGCAGGGTC-3'
mouse-Rb1	Forward primer:	5'-TGCATCTTTATCGCAGCAGTT-3'
	Reverse primer:	5'-GTTCACACGTCCGTTCTAATTTG-3'
mouse-Kdm6b	Forward primer:	5'-TGAAGAACGTCAAGTCCATTGTG-3'
	Reverse primer:	5'-TCCCGCTGTACCTGACAGT-3'
mouse-Pot1a	Forward primer:	5'-TTGGTTTCAACAGCTCCCTATAC-3'
	Reverse primer:	5'-GGAGGGCTTCATAGTTTCCACT-3'
mouse-Atm	Forward primer:	5'-GATCTGCTCATTTGCTGCCG-3'
	Reverse primer:	5'-GTGTGGTGGCTGATACATTTGAT-3'
